# Impact of COVID-19 on Adolescent HIV Prevention and Treatment Services in the AHISA Network

**DOI:** 10.1007/s10461-022-03959-0

**Published:** 2022-12-27

**Authors:** Charisse V. Ahmed, Merrian J. Brooks, Stephanie M. DeLong, Brian C. Zanoni, Irene Njuguna, Kristin Beima-Sofie, Dorothy E. Dow, Aisa Shayo, Alana Schreibman, Jennifer Chapman, Lydia Chen, Shreya Mehta, Michael T. Mbizvo, Elizabeth D. Lowenthal

**Affiliations:** 1 University of Pennsylvania School of Nursing, Philadelphia, PA USA; 2 Department of Pediatrics, University of Pennsylvania Perelman School of Medicine, Philadelphia, PA USA; 3 Children’s Hospital of Philadelphia Global Health Center, Philadelphia, PA USA; 4 Department of Epidemiology, Johns Hopkins Bloomberg School of Public Health, Baltimore, MD USA; 5 Departments of Medicine and Pediatric Infectious Diseases, Emory University School of Medicine, Atlanta, GA USA; 6 Children’s Healthcare of Atlanta, Atlanta, GA USA; 7 Kenyatta National Hospital, Research and Programs, Nairobi, Kenya; 8 Department of Global Health, University of Washington, Seattle, WA USA; 9 Department of Pediatrics, Duke University Medical Center, Durham, NC USA; 10 Duke Global Health Institute, Durham, NC USA; 11 Kilimanjaro Christian Medical Centre, Moshi, Tanzania; 12 Kilimanjaro Christian Medical University College, Moshi, Tanzania; 13 University of Pennsylvania, Philadelphia, PA USA; 14 Population Council, Lusaka, Zambia; 15 Department of Biostatistics, Epidemiology and Informatics, University of Pennsylvania Perelman School of Medicine, Philadelphia, PA USA; 16 CHOP Roberts Center for Pediatric Research, 734 Schuylkill Ave, 19146 Philadelphia, PA USA

**Keywords:** COVID-19, Africa, implementation science, survey, HIV prevention, HIV treatment

## Abstract

We investigated perceived impacts of COVID-19 on the delivery of adolescent HIV treatment and prevention services in sub-Saharan Africa (SSA) by administering a survey to members of the Adolescent HIV Prevention and Treatment Implementation Science Alliance (AHISA) from February to April 2021. We organized COVID-19 impacts, as perceived by AHISA teams, under three themes: service interruptions, service adjustments, and perceived individual-level health impacts. AHISA teams commonly reported interruptions to prevention programs, diagnostic testing, and access to antiretroviral therapy (ART). Common service adjustments included decentralization of ART refills, expanded multi-month ART distribution, and digital technology use. Perceived individual-level impacts included social isolation, loss to follow-up, food insecurity, poverty, and increases in adolescent pregnancies and sexually transmitted infections. The need for collaboration among stakeholders were commonly cited as lessons learned by AHISA teams. Survey findings highlight the need for implementation science research to evaluate the effects of pandemic-related HIV service adaptations in SSA.

**Supplementary Information:**

The online version contains supplementary material available at 10.1007/s10461-022-03959-0.

## Introduction

The dire impacts resulting from the COVID-19 pandemic, caused by severe acute respiratory syndrome coronavirus 2 (SARS-CoV-2), are unequivocal. The compounding socioeconomic ramifications of the pandemic are substantial as SARS-CoV-2 containment measures led to social isolation, increased food insecurity, poor access to healthcare services, loss of financial stability, and other evolving impacts with residual implications [1–4]. Despite a rapidly growing body of COVID-19 literature, there are knowledge gaps regarding the pandemic’s impact on HIV treatment, prevention, and service delivery across sub-Saharan Africa (SSA). In this manuscript we describe COVID-19-related impacts on adolescent HIV service delivery as experienced by AHISA teams. We also discuss how implementation science (IS) can guide next steps in accelerating the pandemic recovery for adolescents living with and at risk for HIV.

Currently, 67% of the global population of people living with HIV reside in SSA [5]. Given the high burden of HIV within SSA, it is crucial to understand the pandemic’s impact on the region’s capacity to concurrently manage both public health threats. The HIV burden in SSA is particularly staggering among adolescents aged 10 to 19 years. In 2016, 2.1 million adolescents were living with HIV (ALHIV), with over 80% residing in SSA [6]. SSA also accounts for 90% of the HIV-related deaths among younger (10 to 14 years) ALHIV worldwide [6]. Consistent adherence to antiretroviral therapy (ART) aided by adequate psychosocial support and uninterrupted ART supply is essential for sustaining viral suppression and thereby preventing HIV-related morbidity, mortality, and HIV transmission among all age groups [7–9]. However, pre-pandemic ART adherence patterns among ALHIV were already worse than for other age groups [10, 11], making ALHIV particularly vulnerable amid the COVID-19 crisis. ALHIV in SSA and across the globe have historically had poor outcomes across the HIV care continuum [12–14], including low rates of viral suppression, high susceptibility to viral rebound, and poor rates of retention when compared to children and adults [15–20]. Without sustained viral suppression, ALHIV may risk immunosuppression and subsequent susceptibility to a host of illnesses, including high symptom severity or even death resulting from COVID-19 [21].

Although the reported burden of SARS-CoV-2 has been relatively low in SSA compared to other regions [22], pandemic-related service interruptions, including drug supply delays and shortages, impede the continuity of HIV treatment and prevention services. In a modeling analysis conducted by Hogan and colleagues [23], HIV-related deaths are estimated to increase up to 10% over the next five years largely due to ART interruptions caused by COVID-19. Due to the limited health infrastructure within many low- and middle-income countries (LMICs) throughout SSA, managing the HIV crisis concurrently with COVID-19 is no small feat. Some LMICs lack robust COVID-19 surveillance structures [24], further hindering opportunities for infection control and disease prevention. These multidimensional factors could complicate the region’s capacity to manage the HIV response particularly among adolescents and other populations that are susceptible to treatment failure. While this is a major challenge, it is also an opportunity for IS since future IS research can inform which system changes that were forced by the pandemic can be best maintained and adapted to lead to care improvements as we emerge from the pandemic.

Since little is known about the impact of COVID-19 on adolescent HIV treatment and prevention services in SSA, we aimed to explore the impact of COVID-19 on the delivery of adolescent HIV treatment and prevention services in SSA countries represented through the Adolescent HIV Prevention and Treatment Implementation Science Alliance (AHISA) Network, a network introduced by the Fogarty International Center in 2017 [25]. AHISA is composed of implementation scientists and their in-country partners from 11 SSA countries and aims to facilitate better utilization of evidence to advance adolescent HIV programming. A total of 26 AHISA teams are conducting collaborative implementation research programs in Botswana (one team), Ghana (one team), Kenya (six teams), Malawi (one team), Nigeria (three teams), Rwanda (one team), South Africa (five teams), Tanzania (two teams), Uganda (two teams), Zambia (two teams), and Zimbabwe (two teams). By evaluating program changes influencing AHISA teams during the pandemic, we provide insights regarding what needs to come next in terms of IS research pertaining to evaluating those changes that are likely to persist post-pandemic.

## Methods

We conducted a survey exploring perceived impacts of the COVID-19 pandemic on adolescent-specific HIV treatment and prevention clinical services and research among AHISA-affiliated projects. AHISA teams conduct IS research related to adolescent HIV. Survey questions were determined by manuscript co-authors, pilot-tested and entered into REDCap [26] for distribution to all 26 AHISA teams. Survey responses from each team’s leader or designee were elicited between February 2021 and April 2021. We requested that each team leader designate a respondent (or responding team) who were knowledgeable about the content of the surveys. The survey included measures of changes in clinical services related to the COVID-19 pandemic. These clinical service changes relate directly to IS since the systemic update of evidence-based practices into routine care are dependent on the clinical context and changes to the clinical context. Items included Likert-scale questions asking teams to describe adolescent HIV clinical care impacts of the COVID-19 pandemic. Surveys included both Likert scale items focused on quantifying changes in needs and availability of services for adolescents (i.e., increased, decreased, or unchanged), and free-text narratives. Teams described: (1) adolescent HIV clinical care impacts of the COVID-19 pandemic, (2) COVID-19 pandemic related changes in adolescent care, and (3) lessons learned. Teams were also asked to outline COVID-19 pandemic related changes in adolescent care at their affiliated sites. Open-ended questions were included to provide additional context and to outline lessons learned.

Quantitative data were outlined using standard descriptive statistics, reporting pandemic-related impacts on adolescent HIV clinical activities. Qualitative responses were analyzed thematically using NVivo 12 (QSR International 2019). To guide our thematic analysis, we developed a posteriori codes related to the pandemic’s impact on service delivery, individual impacts on ALHIV as perceived by AHISA teams, and lessons learned by service delivering entities. We avoid quantifying responses that were obtained as part of the free-text qualitative data since issues mentioned in some qualitative responses (e.g. temporary clinic closures due to staffing limitations) may have occurred at more sites than those that considered the issue salient enough to spontaneously mention as part of open-ended responses.

## Results

Of the 26 AHISA teams, 18 AHISA teams representing South Africa [4], Kenya [3], Nigeria [3], Tanzania [2], Botswana, Ghana, Malawi, Rwanda, Uganda, and Zambia responded to the survey. While each of the 18 teams completed the survey in its entirety, some survey questions were skipped if not applicable to the services provided within a particular team. Therefore, the number of respondents for some items is < 18. Areas addressed are divided below in the following categories: service interruptions, service adjustments, perceived individual-level impacts, and lessons learned.

### Service Interruptions

All AHISA teams that completed the survey indicated that HIV prevention and treatment service availability was substantially hampered by the COVID-19 pandemic. Overall, more than half (10/18) of teams reported periods when certain adolescent HIV prevention and treatment services were completely unavailable due to the COVID-19 pandemic. Table 1 highlights the specific services that were halted at one or more sites. HIV services were sometimes completely unavailable and at other times were restricted and/or delayed by the COVID-19 pandemic.

**Table 1:** Services that had periods of unavailability due to the COVID-19 pandemic

HIV Prevention and Treatment Services Halted by Pandemic	
• STI testing and treatment • Condom and lubrication distribution • Sexual and reproductive health services • HIV testing and counseling • Community-based prevention and outreach services	• Antiretroviral therapy refills • Laboratory monitoring (e.g. viral load testing) • Support group meetings • Psychological counseling • Social work and nutrition services

Some clinics were closed due to staffing shortages or repurposed for COVID-related care. Funding diversion was noted as a contributor to temporary or permanent clinic closures. Clinical funds were diverted to prioritize funding towards COVID-19 services at some sites which meant that patients who had previously received HIV-related care at those sites had to transfer their care to different clinics. According to one team, closure of clinical services due to COVID-19 outbreaks among staff meant that adolescents were “turned away without medication.” Some sites reported that viral load testing was either halted or delayed. One team stated that medication stock-outs led to patients switching regimens or returning more frequently for refills. Halted psychosocial support services included previously available 1:1 counseling in clinics, support groups, and Teen Club sessions. Supplemental clinic-based activities that can be integral to adolescent clinic attendance such as the provision of food, structured group education, and peer interaction were suspended at some sites.

Some AHISA teams reported that HIV-testing and outreach services were either discontinued or limited in access due to COVID-19. One AHISA team noted that community-based outreach prevention and testing services still had not resumed after one year of suspensions. Another concern was that the discontinuation of HIV prevention services negatively impacted some high-risk populations as one AHISA team noted that their HIV testing services historically yielded high positivity rates. The unavailability of outreach services was also a particular concern since they had offered “a refuge for many adolescent men who have sex with men who experience stigma and discrimination in the community” and “supported the healthy social and emotional development of youth.” However, a few AHISA teams mentioned that they attempted to minimize the gap in testing availability by introducing HIV self-testing kits to replace in-person testing. As community-based testing and outreach services were halted during the pandemic, the lack of service availability led to concerns regarding undiagnosed HIV infections among high-risk groups and limited access to condoms.

Table 2 outlines reported changes in service availability. For sexual and reproductive health (SRH) services, there was some heterogeneity between sites. While SRH services were reported as decreased in most locations, one team reported a rise in family planning use among adolescent girls since the pandemic. Reasons for decreases in SRH services at other sites included lockdowns and fear of in-person visits. The pandemic also limited previous efforts to scale up SRH services. Similar to SRH services, there was some heterogeneity with regard to availability of psychosocial support services with most sites reporting decreases, but one site reporting no changes and another site reporting increased availability. The site reporting increased availability of psychosocial support services also reported an increased need with a heightened prevalence of adolescent mental health emergencies.

**Table 2:** Adolescent HIV service availability impacts resulting from the COVID-19 pandemic reported by AHISA teams

Service Availability	Increased	Unchanged	Decreased	Not Applicable or Unanswered
Sexual and reproductive health	1 (8%)	2 (15%)	10 (77%)	5
Preexposure prophylaxis access	0	0	9 (100%)	9
HIV testing	0	0	11 (100%)	7
Linkage to HIV care	0	3 (27%)	8 (73%)	7
HIV-related laboratory monitoring	0	5 (63%)	3 (38%)	10
Psychosocial support services	1 (8%)	1 (8%)	10 (83%)	6

HIV prevention and testing services were decreased at all sites offering these services at baseline. Common reasons for decreases in HIV testing were travel restrictions and prioritization of COVID-19 prevention services such as contact tracing and testing. The perception of HIV risk was also reported to be lower at some sites with reduced sexual encounters due to social distancing. Decreases in linkage to HIV care were attributed to decreases in HIV testing, fear of COVID-19 transmission during travel, and COVID-related travel restrictions. When HIV linkage services were reported to be unchanged, same-day testing and on-site linkage to care played a role in continuity. Laboratory monitoring appeared to be less impacted than other services.

### Service Adjustments

When services were not interrupted, they were commonly adjusted due to pandemic-specific needs. Table 3 outlines service delivery adjustments that were made in response to COVID-19. Decentralizing the distribution of ART refills, extending the period between ART refills, providing clinical services remotely, and remodeling the clinical environment for in-person clinical appointments were most described.

**Table 3:** Common Service Delivery Adjustments

Service Delivery Adjustments reported by AHISA Teams
• Decentralization of antiretroviral therapy (ART) refills
• Multi-month ART distribution (e.g. 2-month refills for unstable groups and 3–6 months for stable groups)
• Virtual support groups through digital technology (e.g. WhatsApp, text messaging)
• Remote clinical appointments by phone
• Remodeling delivery of clinical services (i.e., requiring masks, social distancing, limiting duration of clinic visits)
• Decentralizing clinical service delivery
• Increasing the involvement of peers and community health workers in service delivery

Decentralization of ART refills to community-based distribution sites, such as local pharmacies, minimized travel and in-person clinic time. Several AHISA teams described changes in duration between refills such as providing six months of ART supply, typically referred to as multi-month refills, as opposed to the usual one month. The majority of AHISA teams (n = 12) reported introducing or extending multi-month dispensing. However, the pandemic necessitated resorting to multi-month refills even for patients with poor adherence and high viral loads at some sites. Some teams adjusted the duration of refills based on patient viral status to minimize delays in care for less stable patients (e.g., changing ART delivery to two months among virally unstable patients and three to six months among virally suppressed patients). To address challenges related to in-person clinic visits during the pandemic, one team partnered with a non-governmental organization to provide food and transport reimbursement to encourage adolescents to retrieve their three-month supply of ART refills at the clinic. Another AHISA team reported that community-based ART distribution was introduced for the first time in response to the pandemic. Community ART distribution directly to patient homes was also implemented.

The pandemic necessitated changes from in-person to remote/virtual delivery of numerous psychosocial and clinical services and activities. Some psychosocial support groups, such as Teen Clubs, were shifted to a virtual format due to the pandemic. Digital platforms such as WhatsApp and text messaging have been used to provide social support continuity. Digital platforms were also used for a HIV self-testing verification (i.e. use of remote communication to observe the self-testing process or result). Another service delivery technique adapted since the pandemic was the use of remote service delivery such as phone interviews in lieu of in-person clinic visits. Community-based HIV testing was also introduced as a substitution for clinic-based testing.

In-person clinical services were adapted by reducing frequency of clinical appointments, requiring social distancing in waiting rooms, limiting medication supply and distribution when stock-outs were occurring, and limiting clinical services to patients with severe conditions. Other changes to the clinical milieu included increased use of personal protective equipment, sanitizing stations, infection control signage, and protective screens. Lastly, two AHISA teams highlighted the use of peers to provide continuity in the delivery of adolescent HIV prevention and treatment services. For instance, sexual health peer educators in Kenya were responsible for providing condoms and lubricants as well as tracing and distributing HIV medications to adolescents. Peers were also trained in providing education regarding COVID-19. The pandemic also led to increased engagement of community health volunteers in adolescent adherence and retention support.

### Perceived Individual-Level Impacts

AHISA teams outlined how they believed COVID-19 lockdowns, service restrictions and service adjustments had affected individual-level health outcomes among adolescents, including increased isolation and abuse, food insecurity, poverty exacerbation, mental health concerns, reduced retention in care, medication non-adherence, increases in adolescent pregnancies and sexually transmitted infections, and delayed disclosure of HIV status. AHISA providers feared that limited clinical visits would lead to worsening of HIV disease and increased lost-to-follow-up. Mental health impacts of social isolation among adolescents such as depression and anxiety were also concerns raised by AHISA teams. One team noted anecdotally that adolescent males were most negatively impacted in terms of maintaining viral load suppression and retention in care.

Teams with the ability to quantify changes in clinic attendance and medication adherence were asked to do so and these reports are outlined in Table 4. Common reasons for adolescents missing scheduled clinical visits were reported to be fear of COVID-19 transmission, lack of transportation, and lockdown restrictions. The main contributors to poor adherence were thought to be poor adherence support, decreased income due to COVID-related job loss, lack of public transportation, lockdowns, limited access to ART, and mental health concerns.

**Table 4:** Behavioral impacts resulting from the COVID-19 as reported by AHISA teams

Behavioral Risk Factors	Increased	Unchanged	Decreased	Not Applicable or Unanswered
Poor HIV treatment adherence	5 (55.6%)	4 (44.4%)	0	9
Missing scheduled clinical visits	9 (90%)	1 (10%)	0	8

### Lessons Learned

AHISA teams, when addressing the lessons they learned while adapting to the pandemic, stressed many factors that relate directly to IS such as outer context, inner context, and innovating factors. In terms of the outer context, teams mentioned the need for collaboration and continual engagement with community-level stakeholders and implementers to minimize pandemic effects and maximize continuity of care and support for adolescents and adolescent-based HIV prevention and care services. Inner context factors; including the need for flexibility, adaptability, creativity, and patience among members of the clinical workforce; were noted to be critical to adjusting service delivery to meet the challenges of the pandemic. Innovation factors included different models of remote service delivery. Even with hastily planned transitions that could not benefit from extensive exploration and preparation phases, implementation of remotely delivered services was found to be largely successful. However, there were challenges to reaching some adolescents which necessitated adaptations such as altering the timing of calls. Phone delivery was stated as a potential long-term sustainable alternate strategy for developing stronger patient-provider relationships.

AHISA teams also expressed learning from adolescents themselves to make appropriate modifications. One team stated that they “learn from young people to adapt strategies to meet their dynamic needs and environment.”

## Discussion

Our cross-programmatic evaluation provides insights into the adolescent-focused HIV prevention and treatment services in the AHISA network that were interrupted within the first year of the COVID-19 pandemic as well as adjustments utilized to minimize service interruptions and combat the challenges of an ongoing pandemic. While the current data do not allow us to evaluate the effectiveness of the adapted services, they provide insights into the types of IS research questions that will need to be addressed in the coming years as we emerge from the pandemic and attempt to close the gap in adolescent HIV outcomes. The survey data revealed that COVID-19 interrupted adolescent-focused efforts throughout the HIV treatment cascade, interrupting pre-pandemic progress. However, at the same time, innovative care delivery strategies were rapidly implemented. Systematic IS studies of what worked and what didn’t work are likely to accelerate future progress.

HIV testing services were commonly halted or severely limited to give priority to COVID-19 testing and tracing efforts. Likewise, funding mechanisms to support HIV treatment services were often diverted to support the COVID-19 response. Survey findings also highlight interruptions to ART supply which have serious implications regarding HIV-related mortality. According to recent modelling, a six-month interruption of ART supply in 50% of people living with HIV in SSA can lead to a 1.63- and 1.19-times increase in HIV-related mortality and new HIV infections, respectively [27]. Thus, the incidence of new HIV infections may have been higher at the same time that access to testing and linkage to care was more limited. If this is the case, rapid implementation of evidence-based practices will be necessary over the coming years to avoid additional rises in HIV incidence and mortality in vulnerable young people.

Access to psychosocial supports were also more limited at most sites for ALHIV during the early pandemic period. For example, interruptions in Teen Clubs, a common peer-based differentiated service approach, during the COVID-19 pandemic were common. This was similarly reported in a published paper from Namibia which described Teen Clubs being limited to meeting in places where physical distancing is possible, with no more than 10 adolescents participating at a time [28]. Prior to the pandemic, Teen Club meetings were also when adolescent participants received ART refills [29–32]. AHISA teams reported that medication dispensing shifted to localized community-based ART distribution sites to mitigate the need to travel for ART refills during the pandemic. However, since these distribution sites are open to the public, adolescents may choose to avoid picking up their medications due to fear of stigmatization, further limiting their capacity to maintain optimal adherence. These changes in care delivery approaches raise the need for IS research to evaluate how to most effectively decentralize both psychosocial support and treatment services for ALHIV. Since stigma and lack of peer support predict non-adherence for ALHIV in SSA [33], it is crucial to develop additional implementation strategies to address these concerns as we adapt to the realities of COVID-19 and the post-COVID-19 era. For instance, enlisting peer health workers to deliver community-based services such as ART distribution may address concerns regarding ART refills at decentralized sites and sustain peer-based psychosocial support for adolescents. However, these approaches need to be rigorously evaluated using IS methods and approaches that will allow for comprehensive understanding of what works and what doesn’t work.

Adaptations made out of necessity during the pandemic, but not yet rigorously evaluated, could directly inform adoption of future implementation strategies to improve and sustain access to HIV care among ALHIV. However, application of cohort studies to assess ongoing needs and IS studies to delineate which new approaches work and which do not, is necessary to guide sustained programmatic changes.

Common COVID-19 service adaptations used at AHISA-affiliated clinical sites included expanded ART refill durations and laboratory monitoring, and decentralized ART refills to community settings. Service adaptations identified by AHISA teams are consistent with previous studies in SSA, which similarly utilized strategies that prioritized patients experiencing treatment failure to minimize clinic flow, community-based distribution of ART, remote delivery of health services (i.e., telehealth), and HIV self-testing kits [28, 34–36]. Multi-month ART dispensing, expansion of telemedicine, and the use of digital applications to provide adolescent support group services have been included in guidance from policymakers, including the U.S. President’s Emergency Plan for AIDS Relief, to minimize COVID-19 transmission risks [37]. Additionally, recent studies report sustained ART treatment in SSA and even expedited access to differentiated service delivery by utilizing strategies like multi-month ART refills [2, 38, 39]. While not limited to ALHIV, a time series analysis of the impact of COVID-19 on HIV care in 65 primary care facilities in South Africa revealed that while the pandemic led to decreases in ART initiation and HIV testing, ART provision was generally maintained during the 2020 lockdown periods [40].

AHISA teams reported that some in-person psychosocial support services were adapted for use through mobile technology. Likewise, Hightow-Weidman and colleagues [41] acknowledge the benefit of leveraging the near ubiquitous use of mobile technology to transition to digital interventions during COVID-19; however there may be some limitations to this modality in terms of the IS construct of “reach” for ALHIV in SSA where not all adolescents have smart phones and consistent internet access. Although there is sufficient literature confirming that mobile and electronic health interventions for ALHIV are feasible and acceptable [42–45], evidence regarding the efficacy and effectiveness of these interventions in SSA is lacking. The few randomized controlled trials available display minimal and/or mixed effects regarding the efficacy of mHealth interventions for ALHIV in SSA [46–48], with only a few demonstrating promising effects [49, 50].

Although survey results highlight the limited opportunities for psychosocial support among ALHIV amid COVID-19, the pandemic’s long-term mental health impacts among this population are not yet known. Social isolation, community and interpersonal violence, school absenteeism, and loss of income resulting from COVID-19 have severely affected the mental health of adolescents [51]. Though data are sparse, it is likely that ALHIV have more pandemic-related mental health problems compared to their seronegative counterparts due to higher pre-pandemic rates [52–54]. Not only is peer interaction an essential element of adolescent emotional and social development, peer interaction and support have positive impacts on adolescent treatment adherence [55, 56]. Therefore, it is crucial to ensure the effective delivery and maintenance of interventions that promote ongoing peer interactions among ALHIV.

A strength of this survey-based study is that we provide insights from diverse multinational teams of experts who are knowledgeable on COVID-19-related changes and whose research involves IS to improve adolescent HIV service delivery. However, there are several limitations to our study, including our small sample size and reliance on anecdotal evidence. Another limitation worth noting is that AHISA teams are not fully representative of regular HIV care delivery systems. The state of COVID-related disruptions at AHISA sites likely underrepresents the extent to which services were disrupted at other sites unaffiliated with the AHISA network since AHISA sites include several well-funded “centers of excellence.”

Outcome data during later periods of the pandemic and post-pandemic, and which include nationally representative samples, will be needed to evaluate long-term effects on service provision. Likewise, issues that AHISA teams raised as concerns, such as possible increases in sexual abuse and teen pregnancies, need to be verified at the patient level. Despite limited verification of data from the COVID-19 pandemic, existing literature highlights that similar findings have been verified in other pandemic situations. For example, during the Ebola epidemic, school closures and other lockdowns led to increases in sexual violence and socioeconomic instability which contributed to higher rates of teen pregnancy [45]. Also, an early study conducted in Kenya showed that COVID-19 containment measures were associated with increases in teen pregnancy [57]. In addition to needing more quantitative data to show the impact of the COVID-19 pandemic on adolescents in SSA, IS research is needed to guide effective program recovery and to evaluate the extent to which care strategies that were introduced out of necessity during the pandemic such as digital health innovations, enhanced use of treatment supporters, and cash transfers for food provision [58] should be extended to improve future outcomes.

## Conclusion

While children and adolescents are at lower risk for severe disease and mortality caused by SARS-CoV-2, mitigation and containment measures to combat the COVID-19 pandemic introduced both challenges and opportunities for adolescent HIV service delivery in SSA. COVID-19 significantly disrupted service delivery for ALHIV in SSA, which may have resulted in some adolescents being left behind. However, the responses to those disruptions have resulted in service innovations that may expand health system delivery strategies for ALHIV in the long-term while also illustrating ways rapid service implementation can occur. These innovations may also offer new care models that can increase access to HIV care for adolescents who have difficulties with multiple in-person visits, who were stable and prefer more months of medications, and/or for adolescents who do not utilize the pre-pandemic psychological and social support systems. Future studies must explore how COVID-19 service adjustments have affected HIV-related outcomes of adolescents and the health systems that serve them. Implementation scientists and practitioners can harness COVID-19 service adaptations that were effective and acceptable among ALHIV for further implementation.

## Electronic Supplementary Material

Below is the link to the electronic supplementary material.

Supplementary Material 1

## Data Availability

Not applicable.

## List of abbreviations

AHISA: Adolescent HIV Prevention and Treatment Implementation Science Alliance
ALHIV: adolescents living with human immunodeficiency virus
ART: antiretroviral therapy
COVID-19: novel coronavirus disease 2019
GBV: gender-based violence
HIV: human immunodeficiency virus
IS: implementation science
LMIC: low- and middle-income countries
SRH: sexual and reproductive health
SSA: sub-Saharan Africa
STI: sexually transmitted Infection
